# *Echinococcus granulosus* (*sensu stricto*) (G1, G3) and *E. ortleppi* (G5) in Pakistan: phylogeny, genetic diversity and population structural analysis based on mitochondrial DNA

**DOI:** 10.1186/s13071-020-04199-8

**Published:** 2020-07-13

**Authors:** Mughees Aizaz Alvi, John Asekhaen Ohiolei, Muhammad Saqib, Li Li, Muhammad Haleem Tayyab, Anum Aizaz Alvi, Yan-Tao Wu, Bao-Quan Fu, Hong-Bin Yan, Wan-Zhong Jia

**Affiliations:** 1 State Key Laboratory of Veterinary Etiological Biology, National Professional Laboratory of Animal Hydatidosis, Key Laboratory of Veterinary Parasitology of Gansu Province, Lanzhou Veterinary Research Institute, Chinese Academy of Agricultural Sciences, Lanzhou, 730046 Gansu People’s Republic of China; 2 Department of Clinical Medicine and Surgery, University of Agriculture, Faisalabad, Pakistan; 3 Institute of Pharmacy, Physiology and Pharmacology, University of Agriculture, Faisalabad, Pakistan

**Keywords:** *Echinococcus granulosus*, *Echinococcus ortleppi*, Pakistan, Genetic variation, Haplotype diversity, Phylogeny

## Abstract

**Background:**

Cystic echinococcosis (CE) is a serious tapeworm infection caused by *Echinococcus granulosus* (*sensu lato*) which infects a wide range of animals and humans worldwide. Despite the millions of livestock heads reared in Pakistan, only a few reports on CE prevalence and even fewer on the genetic diversity are available for the country. Meanwhile, the available reports on the genetic diversity are predominantly based on short sequences of the *cox*1 gene.

**Methods:**

To close this knowledge gap, this study was designed to investigate the genetic diversity and population structure of *Echinococcus* spp. in Pakistan using the complete mitochondrial cytochrome *c* oxidase subunit 1 (*cox*1) and NADH dehydrogenase subunit 1 (*nad*1) genes.

**Results:**

Based on BLAST searches of the generated *cox*1 and *nad*1 gene sequences from a total of 60 hydatid cysts collected from cattle (*n* = 40) and buffalo (*n* = 20), 52 isolates were identified as *E*. *granulosus* (*s.s.*) (G1, G3) and 8 as *E*. *ortleppi* (G5). The detection of the G5 genotype represents the first in Pakistan. The phylogeny inferred by the Bayesian method using nucleotide sequences of *cox*1*-nad*1 further confirmed their identity. The diversity indices indicated a high haplotype diversity and a low nucleotide diversity. The negative values of Tajima’s *D* and Fu’s Fs test demonstrated deviation from neutrality suggesting a recent population expansion.

**Conclusions:**

To the best of our knowledge, this report described the genetic variation of *E. granulosus* population for the first time in Pakistan using the complete *cox*1 and *nad*1 mitochondrial genes and confirms *E. ortleppi* as one of the causative agents of CE among livestock in Pakistan. While this report will contribute to baseline information for CE control, more studies considering species diversity and distribution in different hosts across unstudied regions of Pakistan are highly needed.
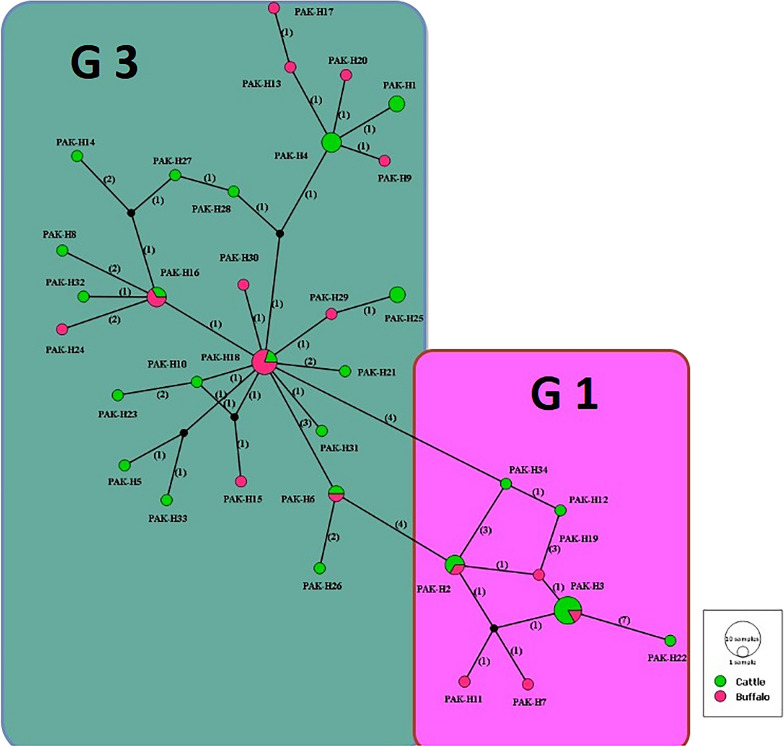

## Background

Cystic echinococcosis (CE) is listed by the World Health Organization (WHO) as a neglected tropical disease having zoonotic connotations [1]. *Echinococcus granulosus* (*sensu lato*) is the etiological agent with a cosmopolitan distribution leading to heavy economic losses amounting for up to 3 billion USD per year [2] and human suffering [3]. Based on nucleotide sequence variation of the mitochondrial DNA, eight different genotypes and *E*. *felidis* within the *E*. *granulosus* complex have been recognized to infect different hosts [4]. *Echinococcus granulosus* (*sensu stricto*) (G1/G3), *E. ortleppi* (G5) and *E. canadensis* complex have the highest significance concerning *Echinococcus* infection in livestock population across the globe [1, 5]. These species require two mammalian hosts to complete their life-cycle *via* a definitive canine host and an intermediate host (domestic or wild ungulate), while humans are considered to be accidental or dead-end hosts [6].

CE is endemic throughout the world including countries in Africa, Europe, South America, the Middle East and Central Asia [7, 8]. Pakistan is an agriculture-dependent country and the livestock sector is an important pillar of the national economy contributing 11.2% to the national GDP [9]. According to the latest livestock census, there are 47.8 million heads of cattle and 40 million heads of buffalo in the country. In the last four decades, there has been no comprehensive data on genetic characterization and molecular diversity of CE in Pakistan. From 1980 to 2015, only three molecular investigations on *Echinococcus* spp. have been reported in animals and were based on partial *cox*1 gene sequences in addition to their limited geographical areas [10].

Given the high population of livestock, scarcity of genetic data and endemic status of CE in the adjoining countries, this study was designed to provide a better insight into the circulating genotypes and to describe the genetic population structure and diversity of *Echinococcus* spp. in animals in Punjab and Khyber Pakhtunkhwa provinces of Pakistan using the full-length *nad*1 and *cox*1 genes.

## Methods

### Study areas

**Fig. 1:**
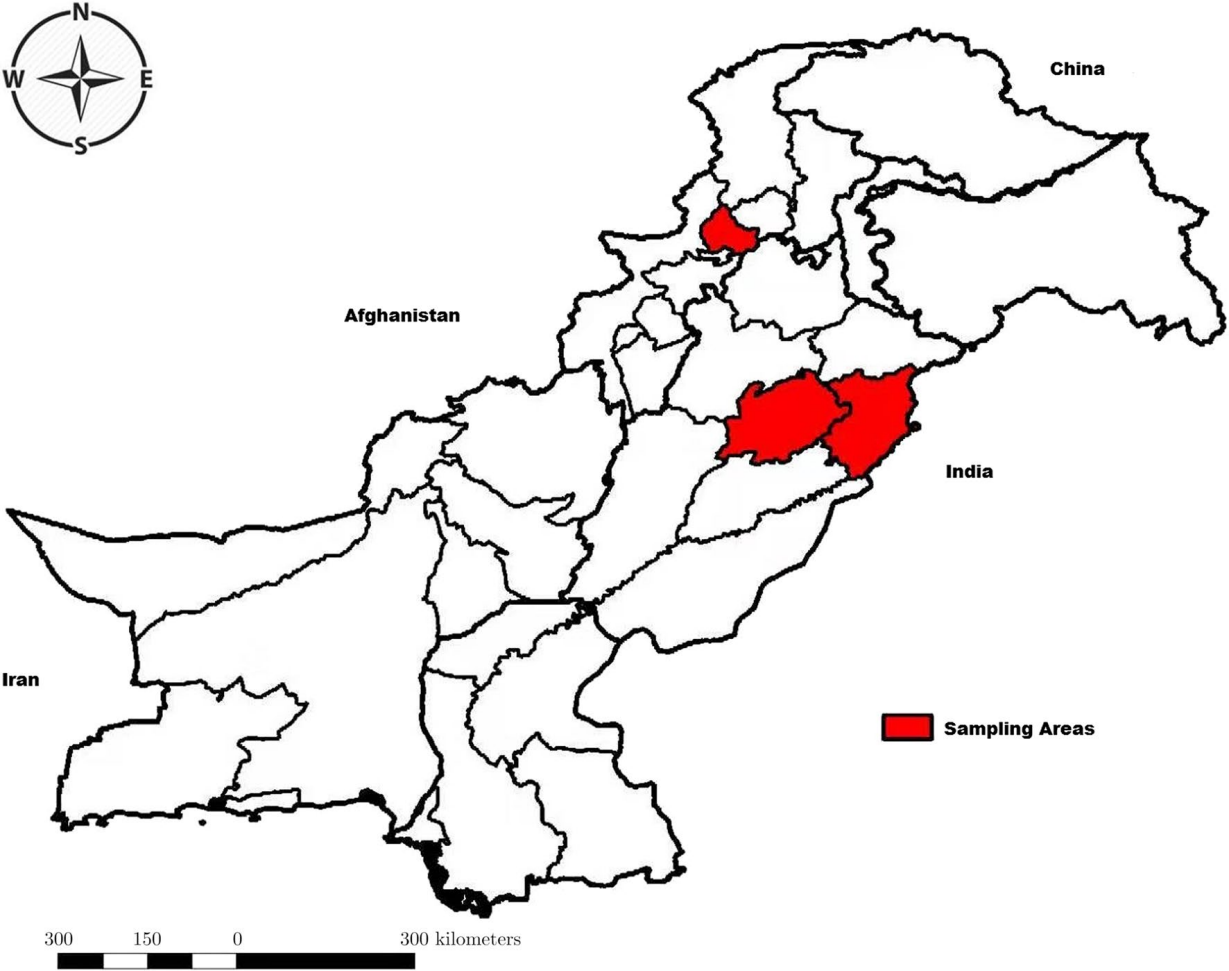
Map of Pakistan. Sampling areas are highlighted in red

Pakistan is located in South Asia bordered by Afghanistan, China, India, and Iran to the west, northeast, east, and southwest, respectively. Hydatid cyst sampling was conducted in Lahore, Faisalabad, and Peshawar (Fig. 1). Lahore is the provincial capital of Punjab and one of the most populous cities in Pakistan. Faisalabad is the third-most-populous city in Pakistan and the second-largest in the eastern province of Punjab. Historically, it is one of the first planned cities within British India and often regarded as the Manchester of Pakistan because of massive industrialization. Peshawar is the capital and largest city of the Khyber Pakhtunkhwa Province.

### Parasite material

Hydatid cysts were collected during September 2019 from abattoirs under the jurisdiction of municipal corporations of Lahore, Faisalabad and Peshawar districts. Before sampling, the stationed veterinary officers were informed about the nature of the study. Inspection of the carcasses was performed, and a total of 60 hydatid cysts were collected. All cysts were of cattle (*n* = 40) and buffalo (*n* = 20) origin. Unfortunately, no cysts were found in sheep and goats slaughtered during this period. The cysts were transported to the Department of Clinical Medicine and Surgery, University of Agriculture, Faisalabad, Pakistan, under refrigerated conditions for further processing.

### DNA extraction, amplification and sequencing of isolates

Hydatid cysts were thoroughly cleaned with 75% ethanol. Germinal layers were washed with phosphate-buffered saline (PBS). Moreover, protoscoleces were repeatedly washed in PBS as described previously [11] and stored until use. DNA extraction was carried out on cut pieces of germinal layers and protoscoleces using the Qiagen Blood and Tissue Kit (Qiagen, Hilden, Germany) according to the manufacturer’s instructions. Amplification of the complete mitochondrial complete *cox*1 gene (1608 bp) using forward primer (5′-ATT ATA GAA AAT TTT CGT TTT ACA CGC-3′) and reverse primer (5′-AAG CAT GAT GCA AAA GGC AAA TAA ACC-3′) and complete *nad*1 gene (894 bp) using forward primer (5′-ATT ATA GAA AAT TTT CGT TTT ACA CGC-3′) and reverse primer (5′-ATT CAC AAT TTA CTA TAT CAA AGT AAC C-3′) [12] was carried out for all isolates. Positive and negative controls were also used. The PCR conditions were carried out as previously described [13] in a final volume of 25 μl consisting of 5 μl of 5× *Taq* buffer, 10 pmol of each primer, 0.2 mM dNTPs, 0.5 μl of Ex *Taq* DNA polymerase (5 U/μl; TaKaRa, Kusatsu, Japan), 2 mM MgCl_2_, 0.5 μl of genomic DNA extract (≥ 20 ng) and RNAse free water to make up the final volume. Amplicons were visualized in a 1.5% (w/v) agarose gel stained with GelRed^TM^ (Biotium, Fremont, USA). Five microliters of the amplicon were used for this purpose while the rest was used for sequencing in an ABI3730Xl DNA Analyser (Beijing Tsingke Biotechnology Co., Ltd., Beijing, China).

### Molecular analysis

DNA sequence alignment and manual examination for misread nucleotide bases were conducted in Unipro UGENE v1.32.0 software [14] while the identity of each isolate was confirmed with their nucleotide sequences using the NCBI BLAST program (https://blast.ncbi.nlm.nih.gov/Blast.cgi).

### Population genetic analysis

Diversity indices such as number of haplotypes (h), haplotype diversity (Hd) and nucleotide diversity (π) were estimated using DnaSP v.6. The neutrality indices (Tajima’s *D* and Fu’s Fs) [15, 16] were estimated in DnaSP v.6 [17]. Median-joining network [18] was inferred based on mitochondrial *nad*1, *cox*1 and *nad*1–*cox*1 gene sequences using PopART (http://popart.otago.ac.nz) for intraspecific data analysis revealing several connections between haplotypes representing possible missing mutational links.

### Phylogenetic analyses

The phylogenetic relationships between haplotypes were inferred by Bayesian method based on the *nad*1–*cox*1 dataset using MrBayes v.3.1.1 [19]. Markov Chain Monte Carlo (MCMC) sampling was used to assess the posterior distribution of parameters with a chain length of 5,000,000 states with parameters logged at every 1000 states and 25% discarded as ‘burn-in’. TreeView v.1.6.6 (http://taxonomy.zoology.gla.ac.uk/rod/treeview.html) was used to display the tree.

## Results

Amplification of the *nad*1 and *cox*1 gene yielded PCR products of approximately 1400 bp and 2000 bp, respectively. Nucleotide sequences of all 60 isolates analyzed in this study were aligned with reference sequences of each genotype within *E*. *granulosus* (*s.l.*) retrieved from GenBank. A total of 3 genotypes of *Echinococcus* were found: G1 (*n* = 14); G3 (*n* = 38); and G5 (*n* = 8). Comparative data on organ location, cysts fertility and genotypes of *E. granulosus* and *E. ortleppi* isolates are shown in Table 1.

**Table 1:** Comparative data on organ location, cysts fertility and genotypes of *Echinococcus granulosus* and *E. ortleppi* isolates

Organ of cyst isolation and fertility	Cattle No. of cysts (genotype count)	Buffalo No. of cysts (genotype count)
Lungs fertile	3 (2 G1, 1 G5)	1 G3
Lungs infertile	32 (5 G1, 23 G3, 4 G5)	15 (4 G1, 11 G3)
Liver fertile	1 G5	2 G3
Liver infertile	4 (2 G1, 2 G5)	2 (1 G1, 1 G3)
Total	40 (9 G1, 23 G3, 8 G5)	20 (5 G1, 15 G3)

### Nucleotide polymorphism

The newly generated sequences of *E*. *granulosus* (*s.s.*) showed a total of 42 mutation sites (*nad*1 = 13 and *cox*1 = 29) with 23 parsimony informative sites (*nad*1 = 8 and *cox*1 = 15). Among the 52 *E*. *granulosus* (*s.s.*) isolates, 12 and 23 haplotypes were found for *nad*1 and *cox*1 genes, respectively while on concatenation of both gene sequences (*nad*1–*cox*1, 2502 bp) 34 distinct haplotypes were identified.

**Fig. 2:**
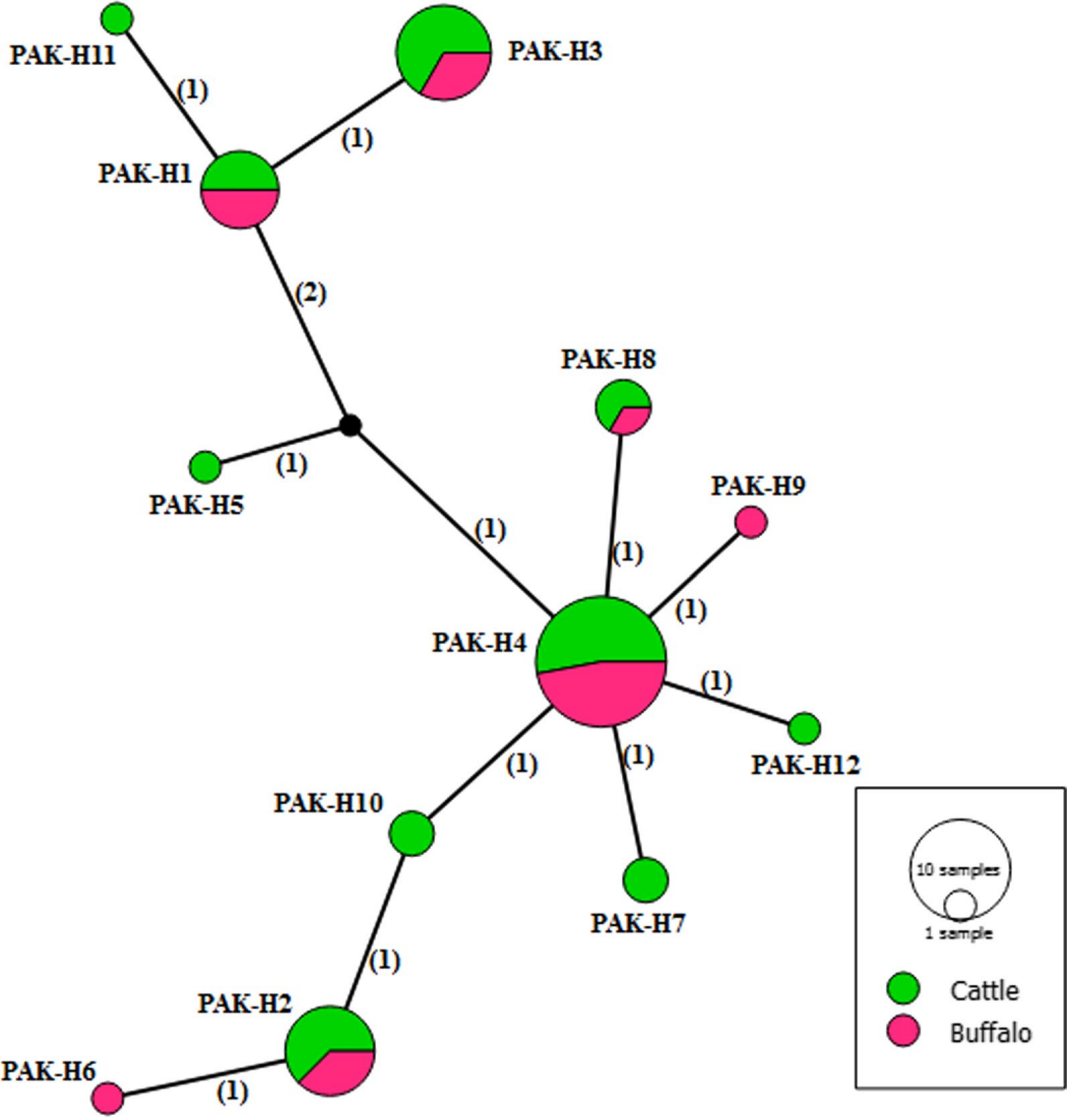
Median-joining network of *Echinococcus granulosus* (*s.s.*) isolates from Pakistan based on complete *nad*1 gene sequences. Circle sizes are proportional to the haplotype frequencies. Numbers in parentheses represent the number of mutations. Small black circles are median vectors

**Fig. 3:**
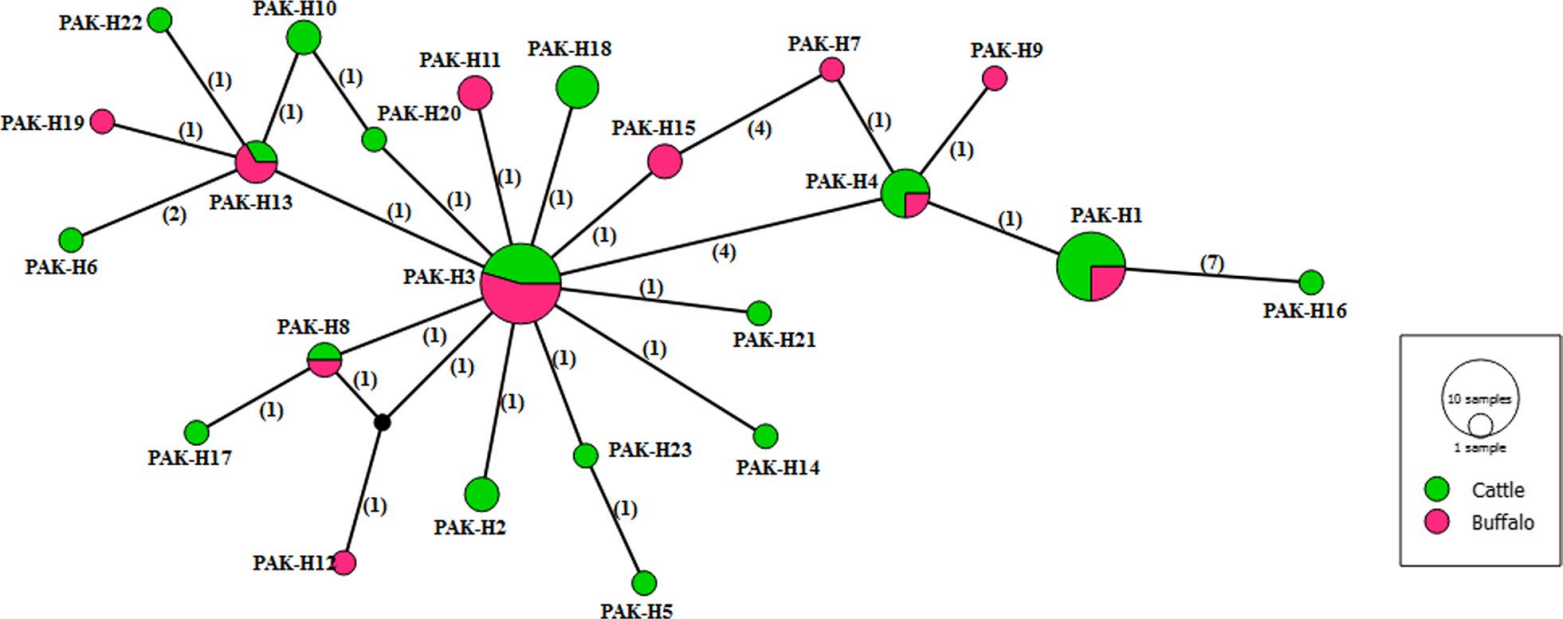
Median-joining network of *Echinococcus granulosus* (*s.s.*) isolates from Pakistan based on complete *cox*1 gene sequences. Circle sizes are proportional to the haplotype frequencies. Numbers in parentheses represent the number of mutations. Small black circles are median vectors

**Fig. 4:**
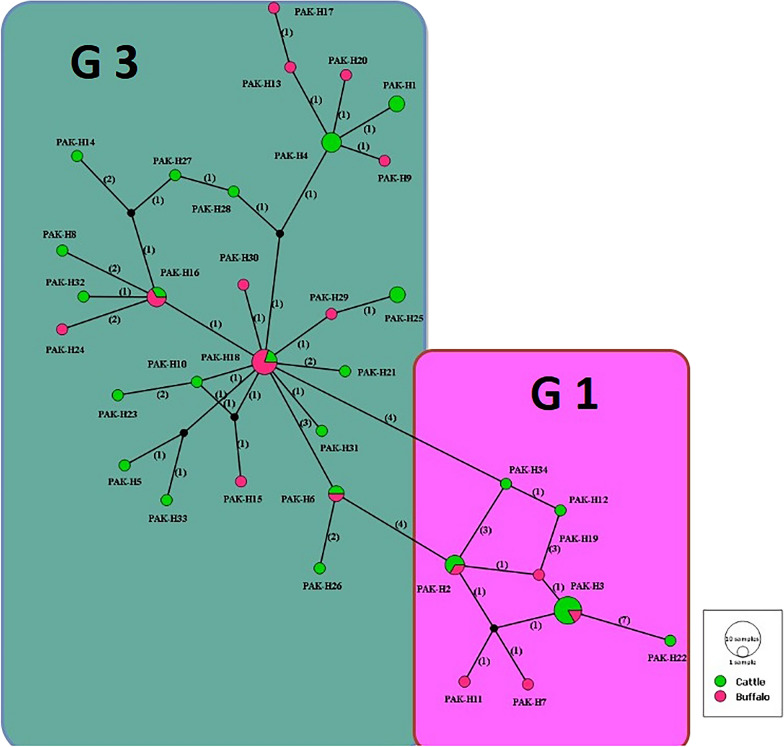
Median-joining network of *Echinococcus granulosus* (*s.s.*) isolates from Pakistan based on complete concatenated *cox*1-*nad*1 gene sequences. Circle sizes are proportional to the haplotype frequencies. Numbers in parentheses represent the number of mutations. Small black circles are median vectors

In the median-joining network for *nad*1 gene sequences, haplotype PAK-H4 had a central position and comprised 32.69% (17/52) of the total population with not more than 4 mutational differences from the other haplotypes (Fig. 2). For the *cox*1 gene, the haplotype PAK-H3 was at the centre of the network, constituting 21.15% (11/52) of the total population with up to 14 mutational differences from the other haplotypes (Fig. 3). Of the 34 haplotypes based on the concatenated sequences of both genes, PAK-H18 was at the centre of the network and constituted 9.61% (5/52) of the total population with a maximum mutational difference of 16 from other haplotypes (Fig. 4). The central haplotype from all three networks comprised isolates from both hosts but did not constitute the majority of the population. The observed nucleotide polymorphism between haplotypes resulted in an amino acid change (see Additional file 1: Tables S1, S2).

Concerning *E*. *ortleppi*, sequence analysis revealed a total of 17 mutation sites (*nad*1 = 5 and *cox*1 = 12) with 11 parsimony informative sites. Out of the 8 samples, 2 and 5 haplotypes were found for *nad*1 and *cox*1 genes, respectively, while 6 distinct haplotypes were observed for the *nad*1–*cox*1 (2502 bp) concatenated sequences dataset.

Representative *nad*1 and *cox*1 haplotype sequences from this study were deposited in the GenBank database under the accession numbers MN886252-MN886293.

### Population indices

The nucleotide diversity and neutrality indices for the entire *E. granulosus* (*s.s.*) and *E. ortleppi* populations were calculated based on the sequences of *nad*1, *cox*1, and *nad*1–*cox*1 genes. For *E*. *granulosus* (*s.s.*), a low nucleotide (π) and high haplotype diversity (Hd) was observed for *nad*1 and *cox*1 genes in both cattle and buffalo. Overall, Hd and π were as follows: *nad*1 (Hd = 0.834, π = 0.00293) and *cox*1 (Hd = 0.925, π = 0.00223) while *nad*1–*cox*1 π and Hd were 0.00248 and 0.972, respectively. Fu’s Fs was negative and insignificant (*P *> 0.05) for the *nad*1 gene. In contrast, it was significant (*P *< 0.05) for *cox*1 and the concatenated *nad*1–*cox*1 sequences. Tajima’s *D* were negative and insignificant for the entire population (Table 2).

**Table 2:** Diversity and neutrality indices for *Echinococcus granulosus* (*s.s.*) populations from Pakistan

Index	*nad*1 (894 bp)			*cox*1 (1608 bp)			*nad*1–*cox*1 (2502 bp)		
	Cattle	Buffalo	Overall	Cattle	Buffalo	Overall	Cattle	Buffalo	Overall
No. of isolates	32	20	52	32	20	52	32	20	52
No. of mutations	11	9	13	24	12	29	35	21	42
Parsimony informative sites	8	6	8	12	9	15	20	15	23
No. of haplotypes	10	7	12	17	11	23	23	16	34
Haplotype diversity (Hd)	0.865	0.805	0.834	0.933	0.900	0.925	0.968	0.963	0.972
Nucleotide diversity (π)	0.00304	0.00287	0.00293	0.00247	0.00185	0.00223	0.00267	0.00222	0.00248
Tajima’s *D*	− 0.01118	0.04283	− 0.26602	− 1.17027	− 0.42821	− 1.45764	− 0.83097	–0.23976	− 1.13030
Fu’s Fs	− 1.672	− 0.453	− 2.207	− 6.720*	− 3.927*	− 11.823*	− 10.559*	− 7.575*	− 21.380*

* Significant (*P *< 0.05)

For *E*. *ortleppi*, overall *nad*1–*cox*1 π and Hd were 0.00257 and 0.893, respectively with insignificant Fu’s Fs (0.032) and Tajima’s *D* (-0.10063) for the entire population (Table 3).

**Table 3:** Diversity and neutrality indices for *Echinococcus ortleppi* population of cattle origin from Pakistan

Index	*nad*1 (894 bp)	*cox*1 (1608 bp)	*nad*1–*cox*1 (2502 bp)
No. of isolates	8	8	8
No. of mutations	5	12	17
Parsimony informative sites	5	11	11
No. of haplotypes	2	5	6
Haplotype diversity (Hd)	0.25	0.857	0.893
Nucleotide diversity (π)	0.0014	0.00322	0.00257
Tajima’s *D*	− 1.5952	0.59845	− 0.1006
Fu’s Fs	2.407	0.867	0.032

### Phylogenetic analysis

**Fig. 5:**
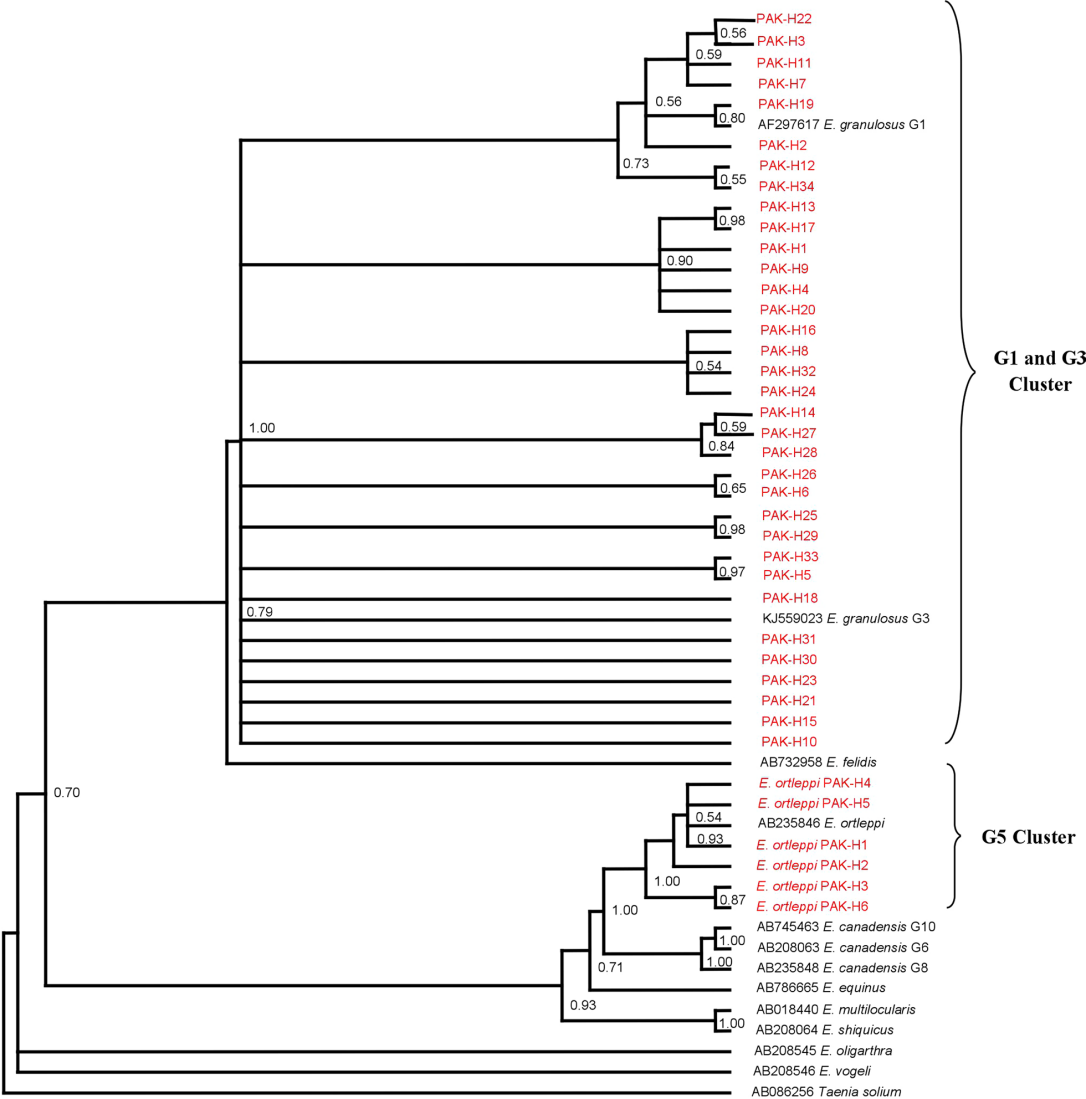
Bayesian phylogeny of Pakistani *Echinococcus granulosus* (G1, G3 and G5) isolates inferred from the *cox*1-*nad*1 (2502 bp) concatenation. *Taenia solium* was used as the outgroup. Red color indicates *E. granulosus* haplotypes isolated in this study. Posterior probability values are depicted at the nodes

The resulting sequences of the different genotypes/species with those retrieved from GenBank were used to construct a phylogenic tree. The Bayesian phylogeny based on a dataset of the concatenated *nad*1–*cox*1 sequences placed all the Pakistani *E. granulosus* (*s.s.*) and *E*. *ortleppi* (G5) isolates in the same clusters with the respective reference genotype sequences from GenBank (Fig. 5). The Bayesian phylogenetic inference confirmed their genotype/species status as distant from other *Echinococcus* species.

## Discussion

Mitochondrial DNA has played an extensive role in investigating intraspecific variations and other population genetic studies because of its conserved structure, maternal inheritance, high mutation rate, absence of recombination, and high evolutionary rate [20–22]. In this study, we report the genetic diversity of *E. granulosus* (G1/G3) and *E. ortleppi* (G5) isolates from buffalo and cattle collected from different slaughterhouses in two provinces of Pakistan based on the complete sequences of two mitochondrial genes, *cox*1 and *nad*1. Previously, in Pakistan, the genetic variation and diversity of *Echinococcus* spp. have mostly been assessed based on partial gene sequences [10, 23–25].

Based on the complete gene sequences, we found the G3 genotype as the most prevalent genotype (38/60) infecting the Pakistani bovine population followed by G1 (14/60) and G5 (8/60). The results are concordant with a previous study conducted in a neighboring country (India) where the G3 (71.8%) was also found to be the most common genotype [26]. Conversely, in China [27], Iran [28], Turkey [29] and Brazil [30] the G1 genotype is reportedly the predominant genotype with 95.74%, 87.5%, 66% and 77.4% prevalence, respectively.

As far as we know, available data on the genetic diversity of *Echinococcus* in Pakistan has been predominantly based on partial sequences of the *cox*1 gene which limits a thorough comparison of the genotypic diversity scenario in Pakistan with its adjoining countries. The length of the gene under study has been reported to potentially affect the outcomes of genetic diversity investigations [31, 32]. For example, using a fragment of the *cox*1 gene in a study conducted on 223 East European and 89 Italian isolates, 24 and 7 haplotypes, respectively were observed [33], while in another study on 69 Argentine isolates 7 haplotypes were observed [34]. Similarly, an investigation conducted on 112 isolates from the Sindh Province of Pakistan based on partial *cox*1 gene sequence resulted in 5 haplotypes [24]. In the present study, we found 34 haplotypes among the investigated *E*. *granulosus* (*s.s.*) population, based on the concatenated genes. The analysis of both mitochondrial genes (*nad*1 and *cox*1) revealed a considerable high level of genetic variation. Higher haplotype diversity and nucleotide diversity were recorded for *cox*1 and *nad*1 gene, respectively of cattle isolates as compared to buffalo isolates. Overall, the diversity and neutrality indices based on the concatenation of both genes were higher in cattle. The radial population structure from the median-joining network observed in the present study differs from the star-like population structure reported in Argentina [34], China [35, 36] and Tunisia [37] where the G1 genotype was found to be preponderant, whereas it was similar to the population structure of *E. granulosus* (*s.s.*) recently reported in Pakistan where the G3 genotype was found to be preponderant [38].

The G5 genotype has been reported in humans in Argentina, Brazil, China, France, India, Mexico and the Netherlands [31, 39–41] indicating that this is an important genotype in terms of public health significance. In this study, to the best of our knowledge, we confirm for the first time, the presence of the ‘cattle’ strain (G5) of the *E. granulosus* (*s.l.*) complex in central Pakistan. This report is also in line with previous observations regarding the prevalence of *E. ortleppi* (G5) in a few Asian countries. For example, the G5 genotype has been reported in cattle from India, Iran, Bhutan, and Nepal [26, 42–44] and recently in a human from China [41]. Host distribution and prevalence of the G5 genotype are highly variable across the world. *Echinococcus ortleppi* (G5) infecting cattle has been reported from Brazil [45], Uruguay [46], France [39], Italy [5], Sudan [47], Ethiopia [48] and Kenya [49]. Out of eight isolates of G5 collected in this study, five were of lung origin which is in line with a molecular survey conducted in Brazil, which had a prevalence of 43.4% for the G5 genotype in cattle, with most of the isolates found in the lungs [45]. Overall, the genetic diversity and neutrality indices based on the mitochondrial genes (*cox*1 and *nad*1) were relatively similar to the genetic diversity of *E*. *ortleppi* in eastern and southern Africa [50].

The neutrality test based on both genes showed negative insignificant values of Tajima’s *D*, indicating an excess of low-frequency polymorphism which suggests a recent population expansion. The values of Fu’s Fs test were negative and significant for *cox*1 and the concatenated *nad*1–*cox*1 sequences, indicating an excess number of alleles, as would be expected during genetic hitchhiking or a recent population expansion. The overall negative values of both Tajima’s *D* and Fu’s Fs tests also confirm an excess of rare mutations within *E*. *granulosus* (G1/G3) populations. In contrast, the *E. ortleppi* (G5) population showed an insignificant negative Tajima’s *D* and insignificantly positive Fu’s Fs.

In the present study, high haplotype diversity suggests a considerable amount of genetic differentiation between the haplotypes as seen in the median-joining network. These results, i.e. the combination of the low nucleotide and high haplotype diversity, suggest a rapid population spread out from a small population size, as described in a previous study [40].

Phylogenetic analysis of the concatenated gene sequences also confirmed the identity of the isolates from buffalo and cattle as *E. granulosus* (G1/G3) and *E. ortleppi* (G5), respectively as they clustered closely with respective *Echinococcus* species (G1-G10) sequences retrieved from GenBank. The basal site of the tree was occupied by Neotropical species, *E*. *vogeli* and *E*. *oligarthra*. The tree also confirmed the sister relationships between *E*. *canadensis* and *E*. *ortleppi*, and between *E*. *shiquicus* and *E*. *multilocularis* as previously described [51].

## Conclusions

The study demonstrates the preponderance of the G3 genotype among all encountered genotypes/species of *E. granulosus* (*s.s.*) (G1, G3) and *E*. *ortleppi* (G5) and emphasizes the important role of this genotype in the distribution of CE among livestock and possibly in human populations. The detection of the G5 genotype also raises some public health concerns due to the increasing reports of human infections with this genotype. Furthermore, analysis of the complete *nad*1 and *cox*1 gene sequences of *E. granulosus* (*s.s.*) population in Pakistan demonstrated a higher genetic variation than what was previously reported based on partial gene sequences.

## Supplementary information

**Additional file 1: Table S1.***Echinococcus granulosus* (*s.s.*) mitochondrial *cox*1 gene nucleotide sequence polymorphism and corresponding amino acid changes among haplotypes from cattle and buffalo. **Table S2.***Echinococcus granulosus* (*s.s.*) mitochondrial *nad*1 gene nucleotide sequence polymorphism and corresponding amino acid changes among haplotypes from cattle and buffalo. **Table S3.***Echinococcus ortleppi* mitochondrial *cox*1 gene nucleotide sequence polymorphism among haplotypes from cattle. **Table S4.***Echinococcus ortleppi* mitochondrial *nad*1 gene nucleotide sequence polymorphism among haplotypes from cattle.

## Data Availability

All data supporting the conclusions of this article are included within the article and its additional files. Representative nucleotide sequences of *nad*1 and *cox*1 genes from the present study are available in the GenBank database under the accession numbers MN886252–MN886293.

## Abbreviations

PBS: phosphate-buffered solution
PCR: polymerase chain reaction
*cox*1: cytochrome *c* oxidase subunit 1 gene
*nad*1: NADH dehydrogenase subunit 1 gene
CE: cystic echinococcosis
MCMC: Markov Chain Monte Carlo
Hd: haplotype diversity
