# Supplementary material for: Echinococcus granulosus (sensu stricto) (G1, G3) and E. ortleppi (G5) in Pakistan: phylogeny, genetic diversity and population structural analysis based on mitochondrial DNA
Source: Parasit Vectors. 2020 Jul 13;13:347. doi: 10.1186/s13071-020-04199-8 (PMC7359271; doi:10.1186/s13071-020-04199-8)
Supplement: Supplementary file 1 — Additional file 1: Table S1.Echinococcus granulosus (s.s.) mitochondrial cox1 gene nucleotide sequence polymorphism and corresponding amino acid changes among haplotypes from cattle and buffalo. Table S2.Echinococcus granulosus (s.s.) mitochondrial nad1 gene nucleotide sequence polymorphism and corresponding amino acid changes among haplotypes from cattle and buffalo. Table S3.Echinococcus ortleppi mitochondrial cox1 gene nucleotide sequence polymorphism among haplotypes from cattle. Table S4.Echinococcus ortleppi mitochondrial nad1 gene nucleotide sequence polymorphism among haplotypes from cattle. [file 13071_2020_4199_MOESM1_ESM.docx]

**Additional file 1: Table S1** Mitochondrial *cox*1 gene nucleotide sequence polymorphism and corresponding amino acid changes among *Echinococcus* *granulosus* (*sensu* *stricto*) haplotypes from cattle and buffaloes

**Table S1a** *cox*1 mutation sites

| **Haplotype** | ***cox*1 DNA mutation sites** | | | | | | | | | | | | | | | | | | | | | | | | | | |
| --- | --- | --- | --- | --- | --- | --- | --- | --- | --- | --- | --- | --- | --- | --- | --- | --- | --- | --- | --- | --- | --- | --- | --- | --- | --- | --- | --- |
|  | **47** | **51** | **58** | **79** | **96** | **105** | **159** | **170** | **174** | **189** | **325** | **421** | **429** | **547** | **633** | **672** | **759** | **810** | **817** | **830** | **855** | **1001** | **1059** | **1171** | **1478** | **1536** | **1592** |
| **PAK-H1** | A | C | G | A | G | A | T | A | T | C | A | A | T | A | C | T | A | C | T | T | T | T | A | A | T | C | C |
| **PAK-H2** |  |  |  |  |  |  |  |  |  |  |  |  |  |  |  |  |  | T |  |  | C | C |  |  | C | T | T |
| **PAK-H3** |  |  |  |  |  |  |  |  |  |  |  |  |  |  |  |  |  | T |  |  | C | C |  |  |  | T | T |
| **PAK-H4** |  |  |  |  |  |  |  |  |  |  |  |  |  |  |  |  |  |  |  |  | C |  |  |  |  |  |  |
| **PAK-H5** |  |  |  |  |  |  |  | G |  |  | G |  |  | G |  |  |  | T |  |  | C | C |  |  |  | T | T |
| **PAK-H6** |  | T |  |  |  |  |  |  |  |  |  |  |  |  |  |  |  | T | C |  | C | C |  |  |  | T | T |
| **PAK-H7** |  |  |  |  |  |  |  |  |  |  |  |  | G |  |  |  |  |  |  |  | C |  |  |  |  |  |  |
| **PAK-H8** |  |  |  |  |  |  |  |  |  |  |  | G |  |  |  |  |  | T |  |  | C | C |  |  |  | T | T |
| **PAK-H9** |  |  |  |  |  |  |  |  |  |  |  |  |  |  | T |  |  |  |  |  | C |  |  |  |  |  |  |
| **PAK-H10** |  |  |  |  |  |  |  |  |  |  | G |  |  |  |  | A |  | T |  |  | C | C |  |  |  | T | T |
| **PAK-H11** |  |  |  |  |  |  |  |  |  |  |  |  |  |  |  |  |  | T |  |  | C | C |  | G |  | T | T |
| **PAK-H12** |  |  |  |  |  |  |  |  |  |  |  | T |  |  |  |  |  | T |  | C | C | C |  |  |  | T | T |
| **PAK-H13** |  |  |  |  |  |  |  |  |  |  | G |  |  |  |  |  |  | T |  |  | C | C |  |  |  | T | T |
| **PAK-H14** |  |  |  |  |  |  |  |  |  |  |  |  |  |  |  |  |  | T |  |  | A | C |  |  |  | T | T |
| **PAK-H15** |  |  |  |  |  |  |  |  |  |  |  |  | G |  |  |  |  | T |  |  | C | C |  |  |  | T | T |
| **PAK-H16** |  | T | A |  | T | G | G |  | A | T |  |  |  |  |  |  |  |  |  |  |  |  |  |  |  |  |  |
| **PAK-H17** | G |  |  |  |  |  |  |  |  |  |  | G |  |  |  |  |  | T |  |  | C | C |  |  |  | T | T |
| **PAK-H18** |  |  |  |  |  |  |  |  |  |  | G |  |  |  |  |  | G | T |  |  | C | C |  |  |  | T | T |
| **PAK-H19** |  |  |  |  |  |  |  |  |  |  |  |  |  |  |  |  |  | T |  |  | C | C |  |  |  | T | T |
| **PAK-H20** |  |  |  |  |  |  |  |  |  |  |  |  |  |  |  | A |  | T |  |  | C | C |  |  |  | T | T |
| **PAK-H21** |  |  |  | G |  |  |  |  |  |  |  |  |  |  |  |  |  | T |  |  | C | C |  |  |  | T | T |
| **PAK-H22** |  |  |  |  |  |  |  |  |  |  | G |  |  |  |  |  |  | T |  |  | C | C | G |  |  | T | T |
| **PAK-H23** |  |  |  |  |  |  |  |  |  |  |  |  |  | G |  |  |  | T |  |  | C | C |  |  |  | T | T |

**Table S1b** *cox*1 amino acid substitution

| **Haplotype** | ***cox*1 amino acid substitution** | | | | | | | | | | | | |
| --- | --- | --- | --- | --- | --- | --- | --- | --- | --- | --- | --- | --- | --- |
|  | 16 | 20 | 27 | 57 | 109 | 141 | 183 | 253 | 277 | 334 | 391 | 493 | 531 |
| **PAK-H1** | K | V | I | Y | I | S | I | I | M | V | I | V | A |
| **PAK-H2** |  |  |  |  |  |  |  |  |  | A |  | A | V |
| **PAK-H3** |  |  |  |  |  |  |  |  |  | A |  |  | V |
| **PAK-H4** |  |  |  |  |  |  |  |  |  |  |  |  |  |
| **PAK-H5** |  |  |  | C |  |  | V |  |  | A |  |  | V |
| **PAK-H6** |  |  |  |  | V |  |  |  |  | A |  |  | V |
| **PAK-H7** |  |  |  |  |  |  |  |  |  |  |  |  |  |
| **PAK-H8** |  |  |  |  |  | G |  |  |  | A |  |  | V |
| **PAK-H9** |  |  |  |  |  |  |  |  |  |  |  |  |  |
| **PAK-H10** |  |  |  |  | V |  |  |  |  | A |  |  | V |
| **PAK-H11** |  |  |  |  |  |  |  |  |  | A | V |  | V |
| **PAK-H12** |  |  |  |  |  | G |  |  | T | A |  |  | V |
| **PAK-H13** |  |  |  |  | V |  |  |  |  | A |  |  | V |
| **PAK-H14** |  |  |  |  |  |  |  |  |  | A |  |  | V |
| **PAK-H15** |  |  |  |  |  |  |  |  |  | A |  |  | V |
| **PAK-H16** |  | M |  |  |  |  |  |  |  |  |  |  |  |
| **PAK-H17** | S |  |  |  |  | G |  |  |  | A |  |  | V |
| **PAK-H18** |  |  |  |  |  |  |  | M |  | A |  |  | V |
| **PAK-H19** |  |  |  |  | V |  |  |  |  | A |  |  | V |
| **PAK-H20** |  |  |  |  |  |  |  |  |  | A |  |  | V |
| **PAK-H21** |  |  | V |  |  |  |  |  |  | A |  |  | V |
| **PAK-H22** |  |  |  |  | V |  |  |  |  | A |  |  | V |
| **PAK-H23** |  |  |  |  |  |  | V |  |  | A |  |  | V |

**Additional file 1: Table S2** Mitochondrial *nad*1 gene nucleotide sequence polymorphism and corresponding amino acid changes among *Echinococcus* *granulosus* (*sensu* *stricto*) haplotypes from cattle and buffaloes

**Table S2a** *nad*1 mutation sites

| **Haplotype** | ***nad*1 DNA mutation sites** | | | | | | | | | | | | |
| --- | --- | --- | --- | --- | --- | --- | --- | --- | --- | --- | --- | --- | --- |
|  | 21 | 70 | 117 | 135 | 178 | 378 | 416 | 486 | 540 | 564 | 567 | 630 | 665 |
| **PAK-H1** | C | G | C | A | A | T | T | G | A | A | T | T | C |
| **PAK-H2** |  |  | T | G |  |  |  |  |  | G |  | C |  |
| **PAK-H3** |  |  |  |  |  |  |  |  |  |  |  |  |  |
| **PAK-H4** |  |  |  | G | G |  | C |  |  | G |  |  |  |
| **PAK-H5** |  |  |  |  | G |  |  |  | G | G |  |  |  |
| **PAK-H6** |  |  | T | G | G |  |  |  |  | G | G | C |  |
| **PAK-H7** |  |  |  | G | G | C |  |  |  | G |  |  |  |
| **PAK-H8** |  |  |  | G | G |  |  |  |  | G |  |  | T |
| **PAK-H9** |  | A |  | G | G |  |  |  |  | G |  |  |  |
| **PAK-H10** |  |  |  | G | G |  |  |  |  | G |  | C |  |
| **PAK-H11** | T |  |  |  |  |  |  |  |  |  |  |  |  |
| **PAK-H12** |  |  |  | G | G |  |  | A |  | G |  |  |  |

**Table S2b** *nad*1 amino acid substitution

| **Haplotype** | ***nad*1 amino acid substitution** | | | | |
| --- | --- | --- | --- | --- | --- |
|  | 24 | 60 | 180 | 189 | 222 |
| **PAK-H1** | V | I | I | C | T |
| **PAK-H2** |  | V | M |  |  |
| **PAK-H3** |  |  |  |  |  |
| **PAK-H4** |  | V | M |  |  |
| **PAK-H5** |  | V | M |  |  |
| **PAK-H6** |  | V | M | W |  |
| **PAK-H7** |  | V | M |  |  |
| **PAK-H8** |  | V | M |  | M |
| **PAK-H9** | I | V | M |  |  |
| **PAK-H10** |  | V | M |  |  |
| **PAK-H11** |  |  |  |  |  |
| **PAK-H12** |  | V | M |  |  |

**Additional file 1: Table S3** Mitochondrial *cox*1 gene nucleotide sequence polymorphism among *Echinococcus* *ortleppi* haplotypes from cattle

| **Haplotype** | ***cox*1 DNA mutation sites** | | | | | | | | | | | |
| --- | --- | --- | --- | --- | --- | --- | --- | --- | --- | --- | --- | --- |
|  | 237 | 270 | 473 | 510 | 573 | 633 | 1000 | 1047 | 1150 | 1365 | 1482 | 1597 |
| **PAK-H1** | G | T | A | T | C | C | A | T | T | T | T | G |
| **PAK-H2** | T | C | G | C | T | T | C |  |  | C | C | A |
| **PAK-H3** |  | C | G | C | T | T | C | C |  | C | C | A |
| **PAK-H4** | T | C |  | C | T | T | C | C |  | C | C | A |
| **PAK-H5** | T | C | G | C | T | T | C | C | C | C | C | A |

**Additional file 1: Table S4** Mitochondrial *nad*1 gene nucleotide sequence polymorphism between *Echinococcus* *ortleppi* haplotypes from cattle

| **Haplotype** | ***nad*1 DNA mutation sites** | | | | |
| --- | --- | --- | --- | --- | --- |
|  | 636 | 681 | 711 | 721 | 810 |
| **PAK-H1** | C | T | A | A | T |
| **PAK-H2** | T | C | G | G | C |
